# Bevacizumab: A Rare Cause of Nonischemic Cardiomyopathy

**DOI:** 10.1155/2018/1361326

**Published:** 2018-08-27

**Authors:** Oreoluwa Oladiran, Salik Nazir

**Affiliations:** Reading Hospital, Tower Health System, West Reading, PA, USA

## Abstract

Left ventricular dysfunction is a rare side effect of bevacizumab occurring in 2–4% of cases. We report the case of a 68-year-old woman who presented to the emergency department (ED) with sudden onset of shortness of breath, orthopnea, and paroxysmal nocturnal dyspnea. She was tachypneic and in respiratory distress. Physical examination revealed jugular venous distention, diffuse expiratory wheeze, and bipedal edema. She had been started on bevacizumab for the treatment of hereditary hemorrhagic telangiectasia 1 month prior to presentation. Laboratory tests revealed BNP of 1697 pg/ml with slightly elevated troponin 0.05 ng/ml. Chest X-ray showed interstitial edema with cardiomegaly, and transthoracic echocardiogram showed ejection fraction of 30% with global hypokinesia. Left heart catheterization revealed widely patent coronary arteries. Flash pulmonary edema secondary to acute left ventricular dysfunction in this case was attributed to recent treatment with bevacizumab after ruling out other possible etiologies. This case highlights the importance of early recognition of this rare but potentially reversible side effect of bevacizumab to prevent long-term sequelae.

## 1. Introduction

Bevacizumab is a humanized monoclonal IgG antibody against vascular endothelial growth factor (VEGF) [1]. VEGF plays an important role in developmental angiogenesis [2]. Substantial evidence also implicates VEGF as a mediator of pathologic angiogenesis—a hallmark feature of hereditary hemorrhagic telangiectasia (HHT) [3]. Bevacizumab is approved by the FDA as first-line treatment for a wide range of ophthalmologic diseases and malignancies, including glioblastoma, breast cancer, and metastatic colorectal cancer [4]. In addition, it is also used as off-label treatment of hereditary hemorrhagic telangiectasia-related refractory bleeding [5]. The main cardiovascular side effects of bevacizumab include hypertension, bleeding, and venous and arterial thromboembolism [4]. In about 2–4% of cases, bevacizumab causes reversible cardiomyopathy and left ventricular dysfunction even in patients without preexisting cardiac disease [6]. Prompt recognition and early treatment with guideline-directed medical therapy along with bevacizumab discontinuation are crucial in treating this potentially reversible but fatal side effect.

## 2. Case Report

A 68-year-old woman with past medical history of hypertension, chronic kidney disease stage 3, hyperlipidemia, and hereditary hemorrhagic telangiectasia (HHT) presented to the emergency department with sudden onset of shortness of breath. She also reported chest pain, orthopnea, and paroxysmal nocturnal dyspnea. Review of systems was otherwise unremarkable. Her HHT was previously managed by regular blood transfusions and epsilon-aminocaproic acid. Because of the need for frequent blood transfusion due to persistent epistaxis and gastrointestinal bleeding, she was started on bevacizumab infusion at 15 mg/kg/dose (1150 mg total) by her haematologist a month prior to presentation. Initial vital signs on presentation revealed respiratory rate of 25/min, oxygen saturation of 65% on ambient air, blood pressure of 138/83 mmHg, and pulse rate of 92/min. Physical examination revealed respiratory distress with diffuse wheeze, jugular venous distention, and trace pedal edema. Laboratory tests revealed markedly elevated brain natriuretic peptide (BNP) of 1697 pg/ml (normal 0–100 pg/ml) with initial troponin of 0.05 ng/ml (normal < 0.04 ng/ml). Chest X-ray revealed pulmonary vascular congestion and interstitial edema with mild cardiomegaly. She was immediately placed on noninvasive ventilation and started on intravenous furosemide with quick symptomatic improvement. Transthoracic echocardiogram (TTE) showed ejection fraction of 30% and global hypokinesia (please see Supplementary Materials (available here)). Of note, TTE done three years prior to index presentation showed ejection fraction of 56%. She does not drink alcohol, and her thyroid function and sedimentation rate were normal making other etiologies of acute systolic heart failure such as thyroid disorder, alcoholic, or inflammatory cardiomyopathy less likely.

**Figure 1:**
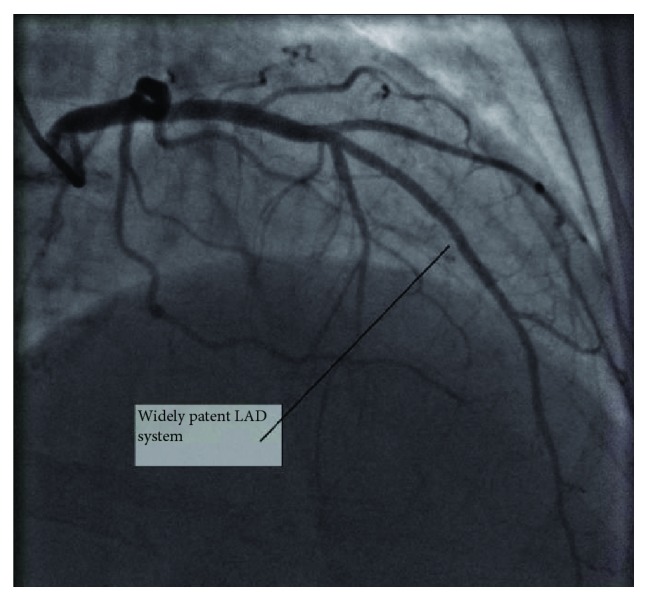


She refused to wear LifeVest and was placed on guideline-directed medical therapy including beta-blocker, angiotensin-converting enzyme inhibitor (ACEi), and aldosterone antagonist along with an oral diuretic. The patient progressively improved and was discharged three days later and scheduled for follow-up with cardiology for outpatient right and left heart catheterization. Two weeks later, she developed another episode of flash pulmonary edema deemed to be due to medication noncompliance. On this occasion, she underwent left and right heart catheterization which revealed widely patent coronary vessels (Figure 1), elevated pulmonary capillary wedge pressure, and elevated left ventricular end-diastolic pressure. She continued to have her monthly bevacizumab infusions with her haematologist as this was not thought to be the cause of her cardiomyopathy at the time. Three months later, she developed sudden onset of chest pain and shortness of breath at home and went into ventricular fibrillation-related cardiac arrest. She underwent prolonged cardiopulmonary resuscitation but eventually had return of spontaneous circulation. She was intubated and admitted to the medical intensive care unit and underwent therapeutic hypothermia. Repeat TTE showed ejection fraction of 34%. She quietly passed away 3 days later.

## 3. Discussion

Drug-induced cardiotoxicity usually manifests as heart failure or left ventricular systolic dysfunction. It is commonly caused by chemotherapeutic medications such as anthracyclines and alkylating agents but is becoming increasingly reported among biologic drugs such as trastuzumab and bevacizumab [7].

Bevacizumab is a recombinant humanized monoclonal IgG1 antibody containing human framework regions and murine complementarity-determining regions. By binding to vascular endothelial growth factor (VEGF), bevacizumab prevents interaction with its receptors (Flt-1 and KDR) on endothelial cell surfaces therefore inhibiting angiogenesis [4]. Bevacizumab has shown promising activity and improve survival of patients with metastatic colon cancer, nonsquamous non-small-cell lung carcinoma, and metastatic breast cancer. Although not FDA-approved, systemic therapy with bevacizumab is a recognized treatment for severe forms of hereditary hemorrhagic telangiectasia (HHT) [5] as was the case in our patient.

Common side effects include bowel perforations, haemorrhage, delayed wound healing, hypertension, and venous and arterial thromboembolism. Left ventricular dysfunction is a rarely reported side effect of bevacizumab with an incidence of 1.2%, and this occurs irrespective of the route of administration [6].

In this case, the probability of causal relationship of acute left ventricular dysfunction and bevacizumab can be rated as certain based on the World Health Organization and the Uppsala Monitoring Centre (WHO-UMC) algorithm [8].

As with other antineoplastic medications, the pathogenesis of left ventricular dysfunction is unknown. Several mechanisms have been proposed including the formation of free radicals in the myocardium and inflammatory cytokines with resultant cardiac depression and congestive heart failure [9]. This side effect appears to be dose dependent, and management involves dose adjustment. Management also involves guideline-directed medical therapy with ACEi/ARBs, beta-blockers, and aldosterone antagonists together with diuretics. Notably patients sick enough to require these medications also have increased risk of toxicity from these medications. Other forms of cardiomyopathies such as Takotsubo cardiomyopathy have also been reported following commencement of bevacizumab [10].

Serial measurement of left ventricular ejection fraction either by transthoracic echocardiogram or by radionuclide angiocardiography, as done in patients receiving doxorubicin, may be a useful modality in monitoring patients planned for bevacizumab therapy via any route while the search for more sensitive and reliable predictors of eventual left ventricular dysfunction continues.

In conclusion, bevacizumab causes reversible dose-dependent left ventricular dysfunction irrespective of the route of administration. It is prudent for clinicians to consider serial transthoracic echocardiogram and monitoring of systolic function to facilitate early detection of this medication side effect and dose adjustment. This is particularly important in patients at high risk of side effects from this class of drugs such as those with low body mass index, advanced age, preexisting or bevacizumab-induced hypertension, and other concurrent chemotherapies [11]. Further research on the pathogenesis of left ventricular dysfunction associated with bevacizumab and the creation of a registry for data synthesis is also needed.

## Supplementary Materials

Supplementary Materials Apical 4-chamber view of transthoracic echocardiogram showing global hypokinesis.
